# Reply to Stein, J.M. Comment on “Onisor et al. Effectiveness and Clinical Performance of Erythritol Air-Polishing in Non-Surgical Periodontal Therapy: A Systematic Review of Randomized Clinical Trials. *Medicina* 2022, *58*, 866”

**DOI:** 10.3390/medicina61091536

**Published:** 2025-08-27

**Authors:** Florin Onisor, Alexandru Mester, Leonardo Mancini, Andrada Voina-Tonea

**Affiliations:** 1 Department of Maxillofacial Surgery and Implantology, University of Medicine and Pharmacy “Iuliu Hatieganu”, 400012 Cluj-Napoca, Romania; florin.onisor@umfcluj.ro; 2 Department of Oral Health, University of Medicine and Pharmacy “Iuliu Hatieganu”, 400006 Cluj-Napoca, Romania; 3 Department of Life, Health and Environmental Sciences, University of L’Aquila, 67100 L’Aquila, Italy; leonardo.mancini@graduate.univaq.it; 4 Department of Dental Materials, University of Medicine and Pharmacy “Iuliu Hatieganu”, 400012 Cluj-Napoca, Romania; andrada.tonea@umfcluj.ro

We thank the authors of the comment [1] for their thoughtful feedback and for engaging with our work. We appreciate this opportunity to respond and to provide further clarification regarding the methodology and conclusions of our article, Effectiveness and Clinical Performance of Erythritol Air-Polishing in Non-Surgical Periodontal Therapy: A Systematic Review of Randomized Clinical Trials [2]. 

The comment raises concerns about our comparison of follow-up values (specifically CAL, PD, and BOP at 6 months) between control and test groups, with the assertion that this does not appropriately measure treatment “efficiency” or “effectiveness,” particularly in light of potentially non-equivalent baseline values. We take this opportunity to explain the rationale and validity behind our analytical choices.

1. Use of Endpoint Values in Meta-Analyses

The central methodological critique involves the use of endpoint (post-treatment) values instead of change-from-baseline scores to assess treatment outcomes. While the concern is valid and reflects a legitimate analytical preference, we respectfully emphasize that the use of endpoint values is a widely accepted and methodologically sound approach in systematic reviews and meta-analyses.

According to the *Cochrane Handbook for Systematic Reviews of Interventions* (Higgins et al., 2022) [3], both endpoint and change scores are valid for meta-analytical comparisons. Specifically,

“Where baseline measurements are similar across intervention groups, meta-analysis of post-intervention values can be as valid as that of change scores.” (Cochrane Handbook, Version 6.4, Section 10.8.2) [4]

In our study, endpoint values were consistently reported across the majority of the included randomized controlled trials (RCTs), while change scores were not uniformly available or reported with adequate statistical parameters (e.g., standard deviations of change). To ensure methodological consistency, comparability across studies, and avoid selective data use, we opted to extract endpoint data as the primary outcome.

This decision was made a priori, documented in our protocol, and transparently stated in the Methods section. Using change scores from only a subset of studies would have introduced additional heterogeneity and potential bias, undermining the internal validity of the meta-analysis.

2. Baseline Imbalances and Randomization

We acknowledge the comment’s point that differences in baseline values can influence post-treatment comparisons. However, the included studies were all RCTs, in which randomization is intended to balance baseline characteristics between intervention and control groups. In our quality assessment, we noted that most trials reported comparable baseline values and demonstrated low risk of bias in allocation and group comparability.

Additionally, any minor baseline discrepancies were considered in our discussion of study heterogeneity and limitations. We also employed a random-effects model, which further accounts for between-study variability and mitigates potential confounding from such baseline differences.

It is worth emphasizing that in clinical practice, the patient’s final periodontal status—not just the magnitude of change—is a key determinant of prognosis and treatment planning. For example, a patient achieving a CAL of 3.0 mm after therapy is clinically better off than a patient improving from 7.0 mm to 5.0 mm, despite there being a greater change in the latter. Thus, final values are highly relevant when assessing clinical effectiveness, particularly in reviews with heterogeneous baseline values.

3. Clarification Regarding Stein et al. (2021) [5]

We thank the authors for highlighting the data from their study. In Stein et al. (2021), the control group had a final CAL of 4.01 mm, and the test group had 4.26 mm, whereas the change from baseline was 0.54 mm and 0.77 mm, respectively. This indeed suggests greater improvement in the test group based on change scores.

However, our analysis did not rely on single-study outcomes to draw conclusions about overall effectiveness. All data were analyzed in aggregate using random-effects meta-analysis. The findings of Stein et al. were integrated along with those from multiple other studies, each contributing proportionally to the pooled estimates. The direction and size of the effect were thus derived from the collective evidence, not individual outcomes.

Importantly, we did not claim that the control group in Stein et al. performed better, nor did we interpret absolute values out of context. We merely reported the standardized findings as part of a consistent methodology applied across all included studies.

4. Terminology: “Effectiveness” vs. “Efficiency”

The comment also challenges our use of the term “effectiveness.” We acknowledge that in the strictest methodological sense, “efficacy” typically refers to performance under controlled conditions, while “effectiveness” refers to real-world clinical outcomes. Our use of “effectiveness” was intended to reflect clinical performance as reported in RCTs with follow-up evaluations—not statistical efficiency, or change-based comparisons.

At no point did we claim universal or categorical superiority of one intervention. Our conclusions were measured and framed within the context of available data, clinical heterogeneity, and study limitations, as explicitly discussed in our manuscript.

5. Conclusion

In summary, we respectfully disagree with the assertion that our use of endpoint data constitutes a methodological error or leads to misinterpretation. Our methodological decisions were consistent with Cochrane-recommended practices, guided by data availability, and transparently documented. While change-from-baseline data are valuable, their inconsistent reporting across studies justified our reliance on endpoint values for analytical integrity and comparability.

We reaffirm the scientific rigor and validity of our meta-analysis and hope this reply clarifies our methodological rationale. We thank the commenters again for their engagement and look forward to continued scholarly dialogue on this important topic in periodontal research.

## References

[1] Stein J.M. (2025). Comment on Onisor et al. Effectiveness and Clinical Performance of Erythritol Air-Polishing in Non-Surgical Periodontal Therapy: A Systematic Review of Randomized Clinical Trials. *Medicina* 2022, *58*, 866. Medicina.

[2] Onisor F., Mester A., Mancini L., Voina-Tonea A. (2022). Effectiveness and Clinical Performance of Erythritol Air-Polishing in Non-Surgical Periodontal Therapy: A Systematic Review of Randomized Clinical Trials. Medicina.

[3] Higgins J.P.T., Thomas J., Chandler J., Cumpston M., Li T., Page M.J., Welch V.A. (2022). Cochrane Handbook for Systematic Reviews of Interventions.

[4] Higgins J.P.T., Thomas J., Chandler J., Cumpston M., Li T., Page M.J., Welch V.A. (2023). Cochrane Handbook for Systematic Reviews of Interventions.

[5] Stein J.M., Yekta-Michael S.S., Schittenhelm F., Reichert S., Kupietz D., Dommisch H., Kasaj A., Wied S., Vela O.C., Stratul S.I. (2021). Comparison of three full-mouth concepts for the non-surgical treatment of stage III and IV periodontitis: A randomized controlled trial. J. Clin. Periodontol..

